# Department of Obstetrics and Gynecology

**Published:** 1893-07

**Authors:** Wm. Keiller

**Affiliations:** F. R. C. S. Ed.; Professor of Anatomy University of Texas; late Physician for Diseases of Women, Edinburgh Provident Dispensary


For Texas Medical Journal.

### OBSTETRICS AND GYNECOLOGY.

By Wm. Keiller, F. R. C. S. Ed., Professor of Anatomy University of Texas; late Physician for Diseases of Women, Edinburgh Provident Dispensary.]

---

CERVICAL DYSMENORRHŒA.—Dr. Hanfield Jones viewing dysmenorrhœa from a clinical standpoint recognizes the following varieties:

1. *Neuralgic.*—Pain accompanies the menstrual flow only when patient's health is deficient, similar to other forms of neuralgia.

2. *Inflammatory.*—This form dates from an attack of pelvic peritonitis or cellulitis. It is greatest during the day or two immediately prior to the period, and is relieved by free hemorrhage.

3. *Membranous.*

4. The group of cases which cannot be classed with the above, and variously called "spasmodic, obstructive, mechanical," the groups under the name *cervical*, because they are associated with abnormal conditions at or near the internal os.

In relation to *cervical dysmenorrhœa* he quotes cases to prove:

a. That rhythmical uterine contractions occur during the early part of menstrnation.

b. That inhibition of the cervical or lower uterine sphincter fibres takes place, and thus dilatation of the cervical canal results.

This dilatation he believes commences some hours before the onset of the flow and is completed during the first day.

Following an analogy between this dilatation and that of the first stage of labor, we know that it is quite common for the cervical canal to be opened up till the os externum presents only a thin ring by painless uterine contractions, and even the whole first stage of labor may be painless. In South African races the whole of the first stage of labor is normally painless. We may therefore suppose that the dilatation in normal menstruation is similarly painless, and further, that those causes which give rise to unusual delay and excessive pain in the first stage of labor will be likely to give rise to painful menstrual dilatation. These causes are .

1st. *Malposition* of the Uterus.—Compare the painful first stage of in an acutely anteflexed uterus with lax abdominal parietes, with the menstrual pain in marked ante and retro displaced uteri. Such cases are relieved by righting the displacement.

2. Muscular spasm of the internal os. This cause, both in labor and menstruation, is removed by full doses of chloral or belladonna. It is probaably not very common.

3. Fibroid thickening at the os internum. Here dilatation gives much relief—drugs are useless.

4. Hyperæsthesia of nerve endings at the internal os. The os is always more or less sensitive to the passing sound, but in some cases the pain is excessive as the sound passes this point. There is usually present endo-cervicitis or endo-metritis, or a history of such a condition which has passed off. The treatment of dysmenorrhœa from this cause is the treatment of the existing inflammation; leeching, salines, iodised phenol, hot douches; or if active inflammation have passed off rapid dilatation will remove the pain.

Lastly, more than one of these causes may co-exist.—*British Medical Journal*, May 27, 1893.

---

Beginning 24 hours after operations involving ligature of a number of the veins of the broad ligaments, and lasting usually for several days, there is commonly an oozing of blood from the uterus. This is not a reflex nervous phenomenon; but simply due to venous congestion, from interference with the venous return, while the arterial supply has not been extensively cut off. In such cases, in spite of removal of the appendages, menstruation will long persist.

In all operations aiming at the production of retrograde changes in the uterus, the operator should endeavor to tie the chief branches of the ovarian arteries, especially when the operation is done for bleeding fibroids. There is no danger of starving the uterus by cutting off too many of its arteries, the danger is all the other way.

---

LIGATURE OF THE BROAD LIGAMENT FOR BLEEDING FIBROIDS.—Dr. Playfair expresses his approval of the vaginal ligature of the broad ligaments for bleeding fibroids, as described by Prof. Martin in the *American Journal of Obstetrics* for April, and thinks that in case where, from the position of the tumor, the uterine arteries would be inaccessible from the vagina, they might be reached by abdominal incision.—*Brit. Med. Journal*, June 10, '93.

Hypertrophic Elongation of the Cervix as a Cause of Obstructed Labor.—Dr. S. Griffith cites two cases. 1. Second labor—cervix three inches long—external os presenting at vulva—cervix admitted finger. Prolonged labor resulting in rupture of the uterus. 2. Multipara—cervix hypertrophied but well dilated. Repeated application of forceps ending in delivery after a very severe and prolonged labor. Metritis and death in five or six days.

Professor Simpson describes a case necessitating embryotomy, and another where free incision of the cervix was resorted to.—*Brit. Med. Journal*, May 6, 1893.

---

Uterine Fibroid in Pregnancy.—Chrobak (*Centralblatl f. Gynak.*, No. 15, '93) removed a uterus at the sixth month of pregnancy, containing a fibroid which weighed five pounds. The tumor contained much necrosed tissue and a cystic cavity. This condition of the tumor explains a method by which fibroids may disappear in pregnancy.

---

Chorea in Pregnancy.—Puech reports a case of a woman, who, in each of her two pregnancies, suffered from chorea, though she had shown no symptoms of it at any other time. It passed rapidly off after labor.—*Archiv. de Tocol. et de Gynec.*, April.

---

Fifty Breech Presentations—All Children Saved.—Etienne publishes these fifty cases, in which, where the children were viable, no child was lost. The rule is routine interference as follows: Leg or trunk born naturally—cord drawn down as soon as it can be reached—as soon as umbilicus reaches vulva, traction is applied, the assistant pressing firmly and constantly on the fundus, to prevent the arms slipping up or extension of the chin. The shoulders are disengaged and the head delivered by two fingers in mouth and other hand over neck. The special maneuvre is the maintainance of pressure on the fundus.—*Arch. dc Tocol. et de Gynecol.*, May, 1893.

---

Early Diagnosis of Uterine Cancer.—Winter laments that three-fourths cases of uterine cancer apply for relief too late. The first sign is free watery discharges, which may last for months before it becomes serious or acquires the characteristic odor. Bleeding is an early sign. It is a most important sign when it occurs after the menopause or in a young woman between the period, after coitus. Pain is a late symptom and seldom appears till after the cellular tissue is invaded. No age is exempt from uterine cancer.—*Rev. Med.* (*Louvain*), March, '93.

